# The impact of grit on adolescent resilience in examining longitudinal mental health outcomes in peri-rural South Africa

**DOI:** 10.21203/rs.3.rs-6558246/v1

**Published:** 2025-05-07

**Authors:** Sahba Besharati, Candice Ramsammy, Furzana Timol, Jeremy Kane, Leslie Davidson, Chris Desmond

**Affiliations:** University of the Witwatersrand; University of KwaZulu-Natal; University of KwaZulu-Natal; Columbia University; Columbia University; University of the Witwatersrand

**Keywords:** Asenze cohort study, adolescence, mental health, resilience, depression, multisystemic

## Abstract

Adolescents in Africa are the world’s fastest growing population group. Despite escalating rates of mental health disorders, little is known regarding the role of protective mechanisms that characterise resilience in adolescent mental health globally and in Africa, where there is heightened exposure to adversities. This study draws on two waves of a longitudinal population cohort from a peri-rural setting in KwaZulu Natal South Africa to investigate the relationship between grit – as a psychological resilience factor - and mental health outcomes in adolescents (N = 1174). Heightened mental health difficulty ratings for internalising factors were found across two study waves, with females reporting significantly higher rates of depression. Grit was found to be significant predictor of lower adolescent depression and anxiety, but dependent on the severity of internalising symptoms, sociodemographic factors and exposure to socioeconomic adversity. Potential differences in the mechanisms of adolescent resilience are highlighted that involve a dynamic interplay between bottom-up and top-down resilience factors in the African context.

## Introduction

Adolescents constitute more than 1.3 billion of the global population^1,2^, with adolescents in Africa representing the fastest growing population group in the world^3^. However, the study of adolescent health and development has only recently gained scientific traction^4,5^. The period of adolescence – roughly defined from 10 to 25 years^6^ – is a unique developmental stage characterised by distinct changes in physical, neurocognitive and social-emotional development^7,8^. These developmental shifts also overlap with a heightened vulnerability to mental health disorders^9,10^. Recent estimates from UNICEF indicate that 20% of adolescents globally experience a mental health disorder^11^ which accounts for at least 13% of the global burden of disease^12^ within the period of early and middle adolescence^6^. Half of adult mental health disorders are also reported to begin during adolescence^9,13^. This escalation in mental health disorders in adolescents has been well documented in high-income counties^9,14^, highlighting the role of early life adversity and wider environmental factors, including social deprivation during the COVID-19 pandemic^15^ and the use of social media^16,17^. However, far less attention has been afforded to the adolescent mental health crisis in low-to-middle income countries (LMICs)^18^ - particularly in Africa^19^. The African context therefore represents both a high adversity context that can result in greater risk for mental health difficulties^20^, as well as an understudied social reality with varying cultural and contextual factors that may uncover different patterns of risk and vulnerability in adolescent mental health globally^7,21^.

Consequently, in line with the 2030 United Nations Sustainable Development Goals^22^, action is needed in combating adolescent mental health difficulties particularly in Africa. The study of adolescent resilience against negative mental health outcomes in the face of adversity is therefore, a critical and underutilised approach in the current mental health literature^18^. Resilience research is based on the fundamental observation that mental health can be maintained despite heightened exposure to stress or adversity^23^. However, the protective factors underlying resilience are poorly understood, especially during adolescence, as a sensitive neurodevelopmental period. Increased focus on adolescent resilience research is even more urgent in low-resource and high-adversity settings^24^, especially across Africa^25^, which are subject to Majority World biases (i.e., limited research in LMICs that house 90% of the world’s population)^1,26,27^.

A culmination of recent resilience research suggests that protective mechanisms that underpin resilience involve a dynamic interaction of internal processes (e.g., “bottom-up”; emotion regulation)^28^ and external factors (e.g., “top-down”; social support and community structures)^29,30^. Aligned with this perspective, drawing on findings from foundational studies in Sub-Saharan Africa^31^ and using cross-cultural comparisons^24,29^, Ungar and Theron^32^ have proposed a multisystemic framework for understanding resilience that is culturally and contextually sensitive. Within this framework, resilience involves multiple interacting processes at a biological, psychological, social and ecological level.

While recent resilience studies in African adolescents^31^ have highlighted the importance of wider external factors at the social and ecological level, resilience also involves internal factors that function at a biological and psychological level^23,32^ that have rarely been investigated in the African context. Emotional regulation for example has been thought to be a critical neurobiological mechanism underlying resilience, especially important in adolescence, where there are distinct changes in white matter pathways and volume, as well as developmental changes in the prefrontal cortex and limbic system^28,33^. Neuroimaging and experimental studies, have not only shown the importance of executive function (e.g., working memory) in adolescent development^34,35^, but also in relation to adolescent resilience and positive mental health outcomes^15^. These neurobiological factors also interact at a psychological level. Although there is consensus in the literature that resilience itself should not be understood as a stable personality trait^23,28^, individual resilience factors – such as cognitive reappraisal^36^ and grit^37^ – nevertheless form part of the psychological protective factors in Ungar and Theron^32^ proposed multisystemic model that promote positive mental health outcomes.

The concept of grit arises from a much longer historical tradition^38^, but it was first described in the scientific literature by Duckworth^39^ as ‘perseverance and passion for long-term goals’. A recent meta-analysis^37^ of the current literature has challenged traditional conceptions of grit or being “gritty” to extend beyond the original hierarchical two-factor model, namely perseverance and consistency, to include wider psychological constructs such as adaptability^37^ that has been previously implicated in resilience and adolescence research^23,40^. Grit has been widely studied in the education literature as a predictor of academic success^41^, and more recent studies highlight the association between grit and positive mental health outcomes^37^, including internalising symptoms^42,43^ (i.e., depression and anxiety). Interestingly, neuroimaging studies investigating the neuroanatomical correlates of grit^44–46^ have identified structures and networks in the prefrontal cortex, limbic system and white matter pathways, which overlap with neural changes in the adolescent brain^9,35^ and in adolescent resilience^28^. Although studies investigating the relationship between grit and mental health have been conducted in diverse country contexts that include Majority World countries^37^, cultural biases still extend to the study of grit^47^, with further research needed to include African populations and different cultural contexts.

Long-standing birth and population cohort studies in Majority World LMICs^48^ – including those in sub-Saharan Africa^19^ – have played a profound role in understanding and promoting health across the lifecourse^9^. To this end, this study draws on data from the Asenze population cohort study, a prospective longitudinal study based in a peri-rural setting in KwaZulu-Natal South Africa that is characterised as a high adversity context^49^. The aims of this study are twofold. Firstly, to characterise the longitudinal mental health profile of internalising factors (i.e., depression and anxiety) of adolescents living in a high stress and adversity setting (e.g., socioeconomic adversity; increased exposure to crime; high rates of HIV) across two waves of the Asenze cohort study. Second, to investigate if grit as one measure of resilience, predicts positive mental health outcomes of African adolescents. This study also draws on Ungar Theron’s^32^ proposed multisystemic approach that integrates culturally and contextually important factors, specifically sociodemographic characteristics and socioeconomic factors, in investigating adolescence resilience. Based on findings from previous cross-sectional studies, we hypothesised that grit would be a significant predictor of lower adolescent depression and anxiety longitudinally when examining internalising symptoms dichotomously and when using continuous scores. As previous longitudinal studies have reported sex differences in the prevalence of internalising symptoms, we also hypothesised that there would be higher rates of depression reported in female adolescents in the cohort across study waves.

## Results

### Participants

**Figure 1:**
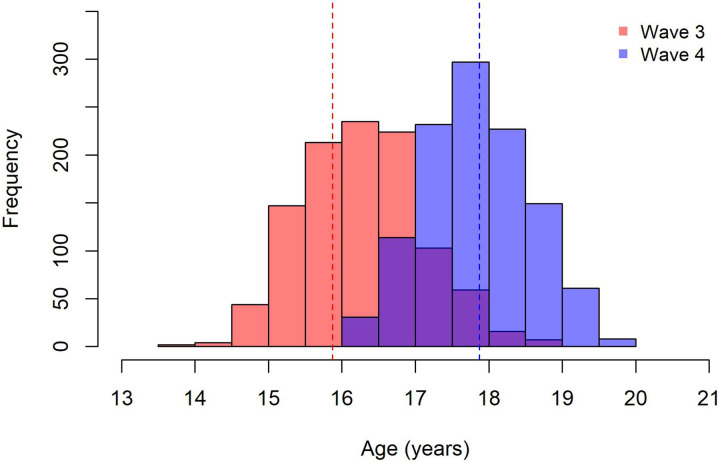
Age distribution of the sample at wave 3 and wave 4. This study used data from the Asenze cohort study, which had completed four waves of data collection at the time of publishing. While the first wave focused on a narrow age range, subsequent waves covered longer timeframes, leading to a wider age distribution in later waves. The histogram illustrates the age distribution of study participants during wave 3 (N = 1174) and wave 4 (N =1120) used in this study. The mean age is indicated by the dotted red line for wave 3 (15.87 [0.92]) and by the dotted blue line in wave 4 (17.87 [0.72]).

We analysed data collected during waves 3 and 4. The cohort contained 1,174 participants in the third wave (average age = 15.87 years; SD = 0.92), of which 1,120 completed the fourth wave (average age = 17.87 years; SD = 0.72; see Table 1 for characteristics and Figure 1 for age distribution across both waves). The proportion of male to female participants remained nearly equal in both waves. By wave 4, a higher number of participants had completed high school, while those remaining in school were predominantly in their senior years (grades 11 and 12). Most participants were HIV negative. Socioeconomic adversity within this sample was assessed during wave 3, characterised by low asset index (24.1%), low caregiver education (32.5%), and food insecurity (11.0%).

The table describes the characteristics of the sample during wave 3 and wave 4. *P*-values refer to the comparison of participant characteristics in wave 3 and wave 4 variables using *Chi-square* tests. The asset index was calculated using a factor analysis model and standardised (mean [SD], 0 [1]) with low assets defined as within the lower tertile, food insecurity refers to answering “often” to any one of the three questions, and low caregiver education was defined as the third quartile of the distribution.

### Longitudinal mental health profiles

**Figure 2:**
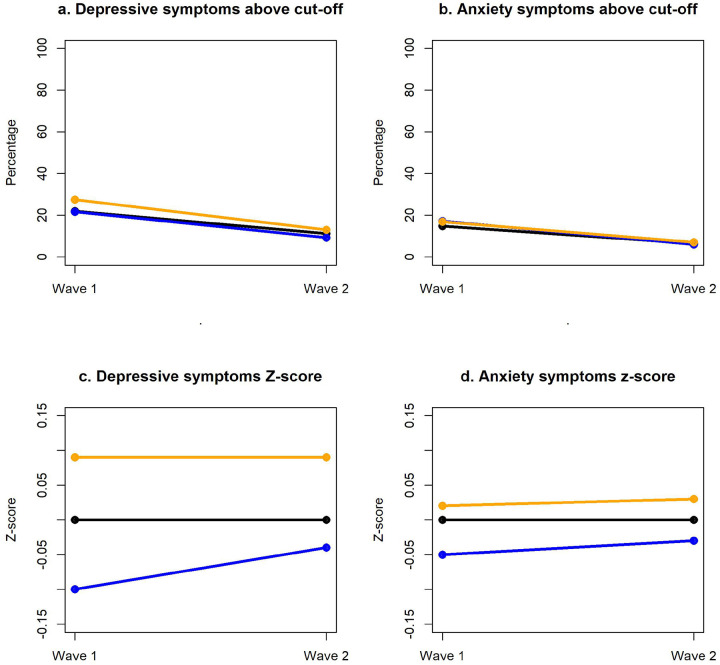
Prevalence of depression and anxiety symptoms amongst adolescents over 2 time points. Depression and anxiety symptom presence and severity was examined by gender. 2a and 2b) Across all participants, 21.9% scored above the depression measure cut-off score and 13% scored above the anxiety measure cut-off scores at wave 3; this reduced to 11.1% and 6.4% respectively in wave 4. 2c) Depressive symptom z-scores across all participants at wave 3 and wave 4; females scored significantly higher at both time points, while males trended towards a higher symptom severity at wave 4. 2d) Anxiety symptom z-scores across all participants at wave 3 and wave 4; there were no differences gender differences in z-scores between wave 3 and wave 4.

First, using dichotomous rating of depression and anxiety as illustrated in Figure 2a, 21.9% of the overall sample presented with depression at wave 3, with a significant decrease to 11.1% at wave 4 [*X*^*2*^ (1, N = 1120) = 32.4, *p* <0.001]. Females were disproportionately affected with a higher prevalence of depression at both waves [*X*^2^ (1, N = 1174) = 6.0, *p* = 0.015 and *X*^*2*^ (1, N = 1120) = 4.0, *p* = 0.046 respectively]. Similarly, 14.7% of the overall sample presented with anxiety at wave 3, with a significant reduction to 6.4% at wave 4 [*X*^*2*^ (1, N = 1120) = 18.7, *p*<*0.001*, *see* Figure 2b]. No gender differences were observed for anxiety in wave 3 or 4 [*X*^*2*^ (1, N = 1174) = 0.001, *p* = 0.981 and *X*^*2*^ (1, N = 1120) = 0.6, *p* = 0.450 respectively]. Additionally, of those that scored above the measures cut-off score at wave 3 for depression (N = 246) and anxiety (N = 163), 21% (N = 52) and 14% (N = 23) respectively, remained above the cut-off score at wave 4. The remaining 58% and 68% of the sample at wave 4 were newly reported cases of depression and anxiety.

In comparison, using continuous scores for depression and anxiety (standardised z-scores; see methods and Figure 2c and 2d), females reported significantly higher depressive symptom severity at both waves [wave 3: t(1049) = −2.55, *p* <0.011, and wave 4: t(1118) = −2.32, *p* <0.020]. While males displayed an increasing trend in depressive symptom severity, the scores were not significantly higher in wave 4 [t(484) = −0.71, *p* = 0.476]. In contrast, no gender differences were observed for anxiety symptom severity [all p’s > 0.360].

### Grit as an individual resilience factor

At wave 3, self-reported grit had an overall mean score of 3.42 (SD = 0.57; range 0–5) indicating average to high levels of grit in the overall sample. Females reported significantly higher levels of grit (means=3.47, SD = 0.59) compared to male adolescents [mean = 3.38, SD = 0.55); *p* = *0.010*; *see supplementary Table 1*]. Grit did not correlate with any indicators of socioeconomic adversity (all *p*’s> 0.05 as shown in supplementary Table 2).

**Figure 3:**
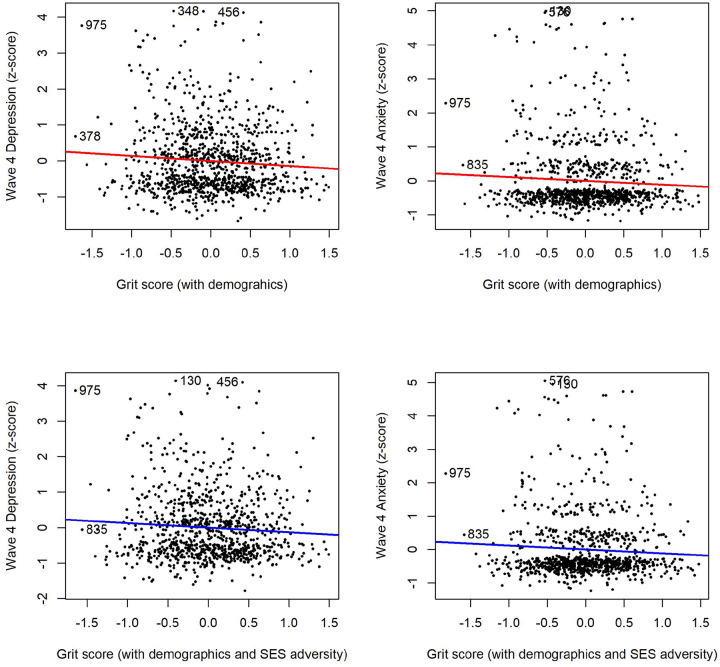
Linear regression analyses to predict depressive and anxiety symptom severity based on grit. Grit scores were regressed onto continuous depressive symptoms z-scores (a) and anxiety symptom z-scores (b) at wave 4. Both models controlled for age, gender, and previous internalising symptoms at wave 3. Both models were significant, with higher grit contributing significantly to lower internalising symptom severity. When adding socioeconomic adversity (low asset index, food insecurity and low caregiver education) alongside the demographic variables (age, gender and previous internalising symptoms), the grit remained a significant predictor for depression (c) but not anxiety (d).

Examining the role of grit in relation to internalising symptom severity, standardised z-scores (continuous scores) for depression and anxiety were used in linear regression models. Higher grit scores were significantly predictive of lower depressive symptom severity [F(1, 1049) = 12.39, *p* <0,001, R^2^=0,01] and lower anxiety symptom severity [F(1, 1049) = 11.54, *p* <0,001, R^2^=0,01; see supplementary Figure 1]. This held true when adjusted for age, gender and previous levels of internalising symptoms as shown in Figure 3a, for both depression severity [F(4, 998) = 19.94, p <0,001, R^2^=0,07] and anxiety severity in [Figure 3b, F(4, 965) = 9.25, *p* <0,001, R^2^=0,04] respectively.

**Figure 4:**
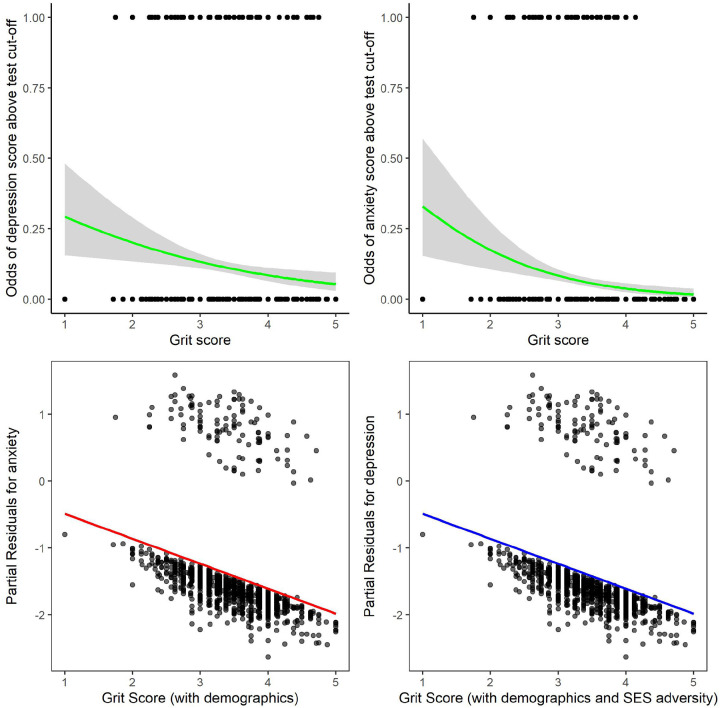
Logistic regression analyses to predict dichotomised rating of depression and anxiety based on grit. Logistic regression analysis showed that grit continued to significantly predict lower depressive symptoms at wave 4 [*X*^*2*^ (1, N = 1051) = 8.30, *p* = *0.004*], with the odds of scoring above the depression measure cut-off score decreasing with every 1-unit increase in grit. In addition, grit significantly predicted lower anxiety symptoms at wave 4 [*X*^*2*^ (1, N = 1051) = 14.50, *p* <*0.001*]. c) When adjusting for adjusted for age, gender and previous levels of internalising symptoms, grit remained as a significant predictor for anxiety [*X*^*2*^ (1, *N* = 1051) = 14.50, *p* <*0.001*] and not depression. The partial residuals shows that grit further reduces the odds of presenting with anxiety after controlling for demographic factors. d) When adding socioeconomic adversity (low asset index, food insecurity and low caregiver education) alongside the demographic variables (age, gender and previous internalising symptoms), grit became a significant predictor for depression [*X*^*2*^ (7, *N* = 968) = 52.13, *p* <0.001], with the partial residuals illustrating that effect. The anxiety model was no longer significant.

To further investigate the role of grit and mental health outcomes, we looked at depression and anxiety scores dichotomously using the measures cut-off scores. Logistic regression analysis showed that grit continued to significantly predict lower depression [*X*^*2*^ (1, *N* = 1051) = 8.30, *p* = *0.004*], with the odds of scoring above the depression test cut-off decreasing with every 1-unit increase in grit as illustrated in Figure 4a. However, grit was no longer a significant predictor of lower depression once the model was adjusted for age, gender and previous depression. When examining anxiety on the other hand, grit significantly predicted lower anxiety [Figure 4b, *X*^*2*^ (1, *N* = 1051) = 14.50, *p* <*0.001*], with this effect remaining significant when adjusting for age, gender and anxiety scores at wave 3 (Figure 4c). The odds of presenting with anxiety above the cut-off decreased for every 1 unit increase in grit scores.

### Socioeconomic adversity, grit and mental health outcomes

Drawing on a multisystemic approach, the regression models were rerun controlling for age, gender and previous internalising symptoms, with the addition of household assets, caregiver education and food insecurity (see supplementary table 3 and table 4 for regression tables). Linear regression results showed that higher grit scores remained a significant predicter of lower depressive symptom severity [Figure 3c, F(7, 960) = 11.08, *p* <0,001, R^2^=0,07]. Although the model was significant when socioeconomic adversity was included, grit was no longer a significant predictor of lower anxiety symptom severity [Figure 3d, F(7, 931) = 5.41, *p* <0.001, R^2^=0,03]. To further investigate the role of grit, logistic regressions were run using dichotomous ratings. Grit became a significant predictor of depression [Figure 4d, *X*^*2*^ (7, *N* = 968) = 52.13, *p* <0.001] when all three indexes of socioeconomic adversity were added to the model. This was not the case for anxiety, as the overall model was no longer significant [*X*^*2*^ (7, *N* = 236) = 13.28, *p* = *0.066*].

### Grit differences for those with high internalising symptoms.

**Figure 5:**
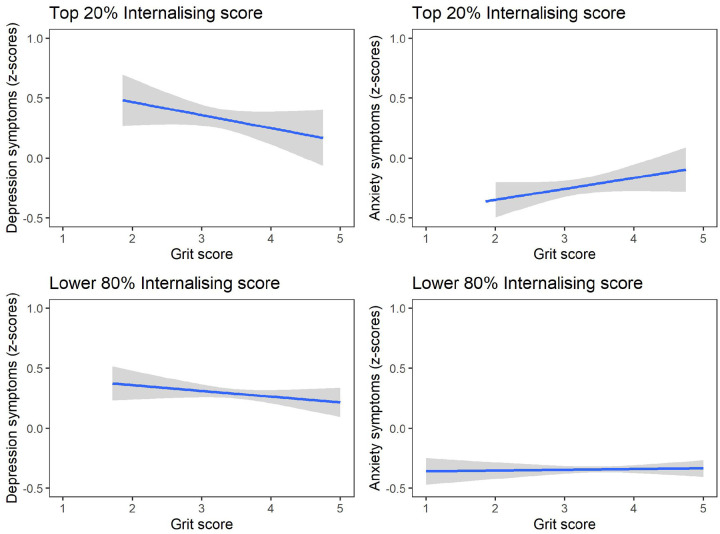
Differential predictive role of grit on internalising symptoms in adolescents across symptom severity. Grit was regressed onto the depressive and anxiety symptoms using z-scores for those presenting with the most severe cases of internalising scores (i.e., in the top 20%) at wave 3, and to the remaining 80%. Linear regression analyses showed that grit was a significant predictor of depressive (a) and anxiety (b) symptom severity in adolescents in the lower 80% range. However, grit was not a significant predictor for lower depressive (c) or anxiety (d) symptom severity in the most severe cases scoring in the top 20%.

Adolescents scoring in the top 20% of the created global score at wave 3 (see methods below) were characterised as those presenting with the high levels of internalising symptoms [N=210; 14.9%] relative to the participants scoring in the remaining 80%. These groups have different patterns of internalising symptoms over time (see supplementary Figure 3). In examining adolescents presenting with the most severe cases of internalising symptoms – in the top 20% at wave 3 – linear regression analyses showed that grit was not a significant predictor of depression and anxiety (all *p*’s > 0.05; see Figure 5). This held true when socioeconomic adversity indicators were included in the model. In the remaining 80% of the adolescent sample however, grit was again predictive of lower depression z-scores [F(3, 849) = 8.365, *p* <0.001, R^2^=0,03], and grit reduced the odds of presenting with depression and anxiety symptoms above the test cut-off scores [*X*^*2*^ (3, *N* = 853) = 25.076, *p* <*0.001* and *X*^*2*^ (13 *N* = 853) = 10.810, *p* = *0.013* respectively]. This once again held true when socioeconomic adversity was added.

## Discussion

Our study provides the first longitudinal evidence of the role of grit as a psychological resilience factor in adolescent mental health globally, and in Africa. More specifically, drawing on data from the Asenze population cohort in a peri-rural context in KwaZulu-Natal, South African adolescents presented with high rates of depression and anxiety across two study waves, with females reporting significantly higher rates of depression. Linear regression analyses confirmed our study hypotheses, with grit consistently predicating lower depression and anxiety when using continues scores and controlling for previous internalising symptoms, gender and age, as well as when adding socioeconomic adversity to the model. However, in the most severe cases of adolescent depression and anxiety (top 20%), grit did not predict lower internalising symptoms. Furthermore, when using a dichotomous categorisation of depression and anxiety a slightly different pattern emerged. Grit predicted lower anxiety and not depression, when controlling for previous levels of internalising symptoms at wave 3, gender and age. In comparison, when socioeconomic adversity was added to the model with dichotomous scores, grit predicted lower depression and not anxiety.

Taken together, these results suggest the differential predictive role of grit as a resilience factor on internalising symptoms in adolescent mental health depending on symptom severity, sociodemographic factors and socioeconomic adversity. The need to assume a multisystemic framework^32^ is therefore further highlighted by our study results. Previous cross-sectional studies conducted in Majority World settings in China^43^ and Thailand^42^ showed similar associations between grit and lower levels of depression and anxiety. However, to the best of our knowledge, this is the first study to demonstrate longitudinal outcomes of grit on depression globally, and in the unique African context, while also focusing on a developmentally sensitive period of adolescence. Furthermore, the use of continuous scores offered a more sensitive analytical approach to changes of internalising symptoms overtime^19^, which also resulted in the most robust results of grit as a significant predictor of lower adolescent anxiety and depression. Nevertheless, our results also highlight that in the most severe cases of mental health difficulties, psychological resilience factors such as grit may not be enough^24,31^, and additional multifaceted interventions are needed^9^. Here, it is important to note that our results are not aligned to neo-liberal views that hold the individual solely responsible for their mental health^50,51^ or that resilience is to be attributed to individual traits alone^40^. Rather, our study provides a new perspective on the role psychological protective factors, such a grit, *within* a multisystemic approach to the study of resilience^32^. Future studies are therefore needed to investigate the early determinants of protective factors like grit and what conditions might foster its development. Similarly, studies should be undertaken to investigate the role of similar psychological factors in resilience such as growth mindset^44,45^ to help uncover the underlying mechanisms involved in the interplay between top-down and bottom-up processes in adolescent resilience. For example, recent findings^52,53^ suggest that psychological resilience factors such as grit, emotion regulation and self-efficacy can be enhanced with resilience-based interventions, however the mechanisms underlying such changes, and the interaction with multisystemic factors, are still unclear.

Furthermore, adolescent girls in this study reported significantly higher rates of grit, but also depression, which is consistent with prior studies showing increased vulnerability to depression in adolescent females^9,54^. These sex differences in depression may also explain why grit was no longer predictive of lower rates of depression in the adjusted model when looking at the dichotomised mental health outcomes for anxiety and depression. Future studies drawing on neuroimaging methods in the African context^21^ could unlock novel insights into the underlying processes involved in potential sex differences or gender-related factors^55^, such as emotion regulation, hormonal changes or psychosocial stressors, in resilience research. For example, a recent protocol study using a longitudinal birth cohort in a peri-urban setting in Cape Town South Africa^56^, will draw on MRI methods to investigate longitudinal neurodevelopmental changes in resilient emotion regulation. Similarly, in looking at the dichotomised internalising scores, the degree of socioeconomic adversity changed the predictive power of grit, with only depression remaining significant in the model. The association between socioeconomic adversity and depression has been well documented in the literature^57^, however future studies are needed to explore the possible moderating relationship of socioeconomic adversity on grit and internalising symptoms. Furthermore, this study drew on objective and rigorous measures of socioeconomic levels – household assets, caregiver education and food security. However, these measures have not always been sensitive enough to capture the complexity of *perceived* socioeconomic inequality^58^, with recent studies advocated for the use of subjective fiscal appraisals^59^.

The longitudinal mental health profile of this sample of South African adolescents is consistent with previous longitudinal studies based in peri-urban settings in South Africa as a Majority World LMIC^19,54^. However, our study is the first to provide longitudinal evidence of high rates of internalising symptoms in South African adolescents in peri-rural settings. The decline in reported anxiety and depression from wave 3 to wave 4 in the study could suggest potential differences in internalising problems in younger compared to older adolescents^19^, or increased stigma^31^ in reporting symptoms in older adolescents within this context. However, this effect might also be attributed to the impact of the COVID-19 pandemic during data collection at wave 3 of the cohort study that ran from 2019 to 2021^49,60^. Data collection was halted for a period due to severe lockdown restrictions at the end of March 2020. The negative effect of the pandemic on adolescent mental health has been robustly shown globally^7^ and even within this study cohort^60^. Therefore, one possibility is that marked higher rates of depression and anxiety were reported post the lockdown period when data collection resumed, which then reduced in data collected at wave 4 in 2022. However, a recent study^61^ examined depression and anxiety symptoms in adolescents in the Asenze cohort throughout wave 3 and found no relationship between internalising symptoms and government-imposed lockdown restrictions. Therefore, although it is unlikely that the COVID-19 lockdown accounted for changes in internalising symptoms in adolescents in the cohort study, future studies should still explore the potential effect of lockdown restrictions during the pandemic as a source of social deprivation^15^ in South African adolescents in the Asenze study and more broadly.

Despite the novelty of our results, our study was not without limitations. The study was bound by the use of psychological measures developed in non-Majority World settings that often lack the cultural sensitivity needed in the current study context^26^, despite language translations used in the current study and South African validation studies of our measures ^e.g.,62–64^. Nevertheless, drawing on dual analytic strategies of using both dichotomous classification and continuous scores that were independent of the tests cut-off measures acted as a further buffer to counteract this limitation. Furthermore, the mental health measures used only included internalising mental health factors. Previous longitudinal studies in South Africa have shown a different pattern of mental health outcomes for male and female adolescents for externalising and internalising factors respectively^19^. Future studies are needed to test if grit is predictive of lower internalising and externalising symptoms and the possible sex differences involved. Lastly, none of the measures used were diagnostic in nature and were all based on self-report methods that are limited by inherent biases involved, which include mental health stigma^31^. Nevertheless, given the lack of longitudinal data on adolescent mental health in culturally diverse context^7^ such as in this cohort study, the use of such screening tools is an acceptable limitation until more formal diagnostic interviews can be utilised^9,65^.

## Conclusion

Housing the largest and fastest growing adolescent population in the world, safeguarding adolescent mental health across the African continent is a critical public health mandate. Resilience research offers an alternative approach to traditional intervention strategies, by investigating the protective mechanisms that promote positive mental health outcomes^18,23^.

This study highlights the importance of adolescence and mental health research conducted in the African context that provides unique insights into the dynamic interplay between neurobiological and psychological protective factors, such as grit, which are embedded within wider socio-cultural processes and ecological structures.

## Methods

### Study setting and population

This study draws on data from the Asenze cohort study, a longitudinal population-based study in KwaZulu-Natal, South Africa. This peri-rural site is characterised by high rates of HIV, food insecurity and unemployment^49,65^. The study follows the health, development, well-being and psychosocial functioning of children. Four waves of data collection occurred from 2008 to 2022: wave 1 (4–6 years old), wave 2 (6–8 years old), wave 3 (13–19 years old), and wave 4 (16–20 years old)^49^. This study draws on data collected in wave 3 (2019–2021) and wave 4 (2022). Data was primarily collected in person for wave 3, with a small portion interviewed telephonically in order to retain those participants who had relocated. Data was collected telephonically in wave 4. Caregiver consent where applicable, and participant assent/consent, were obtained at each wave of the study. Modest attrition was observed with 83.5% of the wave 2 cohort interviewed at wave 3, and 95% of the wave 3 cohort interviewed at wave 4. A fifth round of data collection is currently underway.

Ethical approval was received from the Biomedical Research Ethics Committee of the University of KwaZulu-Natal (BF 036/07 and BE 609/18) and from the Institutional Review Board of Columbia University (IRB No. AAAC2559). Initial approval was also received from local authority councils, the local district health committee, and the local district board of education. Data has been collected on demographic variables, including sex assigned at birth, age, education level completed, HIV status, and socioeconomic variables. Data was collected at wave 3 and 4 on grit, and mental health outcomes amongst others. All material was available in English and Zulu, the languages most used in the area.

### Assessment of mental health: Internalising symptoms

Mental health assessments focused on internalising symptoms - specifically depression and anxiety -that were assessed using validated self-report questionnaires at wave 3 and wave 4.

#### Depression.

During wave 3, the Patient Health Questionnaire-9 (PHQ-9) was used to screen and measure the severity of depression symptoms based on the DSM-5 criteria for major depressive disorder^66^. Participants rated nine items in reference to the past two weeks, using a 4-point Likert scale (0 = not at all; 1 = several days; 2 = more than half the days; and 3 = nearly every day). The scores for each item were summed, resulting in a total score ranging from 0 to 27, with higher scores indicating greater symptom severity. The PHQ-9 has strong psychometric properties (Cronbach’s alpha: 0.71–0.89) and has been validated in multicultural environments, including African contexts^62,63,67^. During wave 4, participants completed the brief PHQ-2, which consists of the first two questions of the PHQ-9. Total scores on both measures ranged from 0–6, with higher scores indicating more greater symptom severity.

#### Anxiety.

At wave 3 Generalized Anxiety Disorder questionnaire- 7 (GAD-7) was used to screen for generalised anxiety disorder according to the DSM-5^68^. The seven items are in reference to the past two weeks and were reported on a 4-point Likert scale from 0 (“not at all”) to 3 (“nearly every day”). The scores were summed, resulting in a total ranging from 0 to 21, with higher scores indicating greater symptom severity. The GAD-7 has been shown to have strong psychometric properties (Cronbach’s alpha: 0.69–0.87), and has been validated in South Africa^62,69^. For wave 4, participants completed the brief GAD-2, which consists of the first two questions of the GAD-7. Total scores range from 0 to 6, with higher scores indicating more frequent symptoms^70^.

Since different depression and anxiety measures were used at each wave, scores were standardised in two ways. First, using the measures cut-off scores, depression and anxiety was looked at dichotomously, with participants scoring above the measure’s cut-off score being categorised as having depression or anxiety respectively. For the PHQ-9 and GAD-7, scores of 10 or higher were categorised as presenting with depression or anxiety symptoms. If lower, it was categorised as minimal or no depression or anxiety symptoms respectively. For the PHQ-2 and GAD-2, scores of three and higher indicated the presence of depression or anxiety symptoms respectively, while lower scores indicated minimal or no symptoms. Dichotomising the scores enabled us to describe them longitudinally. Second, the depression and anxiety scores were standardised (z-score transformed) allowing us to look at the data as a continuous measure of depression and anxiety symptom severity overtime. A global score for internalising symptoms was also created by averaging the depression and anxiety standardised z-score at each wave, with higher scores indicating a greater presence of internalising symptoms (supplementary Fig. 1).

### Assessment of grit

Grit was assessed during wave 3 using the 8-item Short GRIT Scale (GRIT-S)^39,71^. Participants rated how much each statement described them, from 1 (“Not like me at all”) to 5 (“very much like me”). The eight items were averaged to create a total score, ranging from 1 to 5, with higher scores indicating more grit. Although internal consistency was poor in this study (Cronbach’s alpha = 0.297), previous research has demonstrated good validity and reliability globally (Cronbach’s alpha: 0.75–0.81)^72,73^, as well as within South Africa (Cronbach’s alpha: 0.71–0.72)^64,74^.

### Socioeconomic adversity

Drawing on previous methods, socioeconomic adversity was measured using the following indicators: (1) asset index (using household characteristics, assets, and source of heating), (2) low caregiver education (the third quartile of the distribution) and (3) food insecurity (answering “often” to any one of the three questions). The asset index was calculated using a factor analysis model and standardised (mean [SD], 0 [1])^65^. Socioeconomic adversity indicators were dichotomised as follows: low assets (within the third quartile of the distribution), low caregiver education (the third quartile of the distribution), and food insecurity (answering “often” to any one of the three questions: 1) In the past 4 weeks how often was there no food to eat of any kind in your house because of lack of money; 2) In the past 4 weeks, how often did you or any member of your household go to sleep hungry because of lack of food; 3) In the past 4 weeks, how often did you or any of your household go a whole day and night without eating because of lack of food).

### Analysis

Data were analysed using IBM SPSS software version 29.0.2.0, while regression models and figures were generated in RStudio 2024.09.0 + 375. Descriptive statistics were run to characterise the sample, using independent sample t-tests for comparison and chi-square tests for dichotomised variables (i.e., PHQ and GAD variables).

Linear regression models were performed using depressive and anxiety z-scores (continuous scores) at wave 4 as the outcome measure. Grit was the predictor variable, with age, gender, and depressive or anxiety z-scores at wave 3 as covariates. When examining the effects of socioeconomic adversity, grit was the predictor variable, with age, gender, depressive or anxiety z-scores at wave 3, low assets, food insecurity and low caregiver education as covariates. Logistic regressions were performed with the outcome variables being dichotomised depression and anxiety scores at wave 4. Similar to the linear regressions, grit was the predictor variable, with age, gender, depressive or anxiety z-scores at wave 3, low assets, food insecurity and low caregiver education as covariates. Models were performed on the whole sample, as well as a subset of participants who scored in the top 20% of the global internalising scores at wave 3, and with participants who scored in the remaining 80%. *P* values were evaluated using 2-sided 2-sample *t* tests and *χ*2 tests, and significance was set at *P* < 0.05.

Lastly, although the mean age for adolescents in the study at wave 3 and wave 4 were 15.87 and 17.87 years respectively, the age range of the sample includes a much broader range (13 to 20 years across both waves). As previous studies have shown developmental differences according to age distribution, the same set of analysis were run in a subset of the sample to include only 15–17-year-old adolescents across both waves, finding the same pattern of results.

## Figures and Tables

**Table 1.** Sociodemographic characteristics of adolescent participants at wave 3 and 4

	Wave 3^1^	Wave 4^1^	
	N = 1,174	N = 1,120	*p-value*
Age (years)			
Mean [SD]	15.87 [0.92]	17.87 [0.72]	-
Range	13 – 19	16 – 20	
Gender			
Male	586 (49.9%)	546 (48.8%)	0.672
Female	588 (50.1%)	574 (51.2%)	
Attending school			
Yes	1111 (94.6%)	885 (79.0%)	**<0.001**
Completed high school	2 (0.2%)	147 (13.1%)	
Technical and Vocational Education and Training	9 (0.8%)	34 (3.0%)	
No	52 (4.4%)	54 (4.8%)	
Grade			
Lower than Grade 8	23 (2.1%)	2 (0.2%)	**<0.001**
Grade 8	90 (8.1%)	8 (0.9%)	
Grade 9	225 (20.3%)	39 (4.4%)	
Grade 10	384 (34.6%)	195 (22.0%)	
Grade 11	301 (27.1%)	302 (34.0%)	
Grade 12	85 (7.7%)	341 (38.4%)	
Missing	3		
HIV Status			**<0.001**
Positive	83 (7.1%)	88 (8.1%)	
Negative	1082 (92.9%)	996 (91.9%)	
Missing	9	36	
Grit score	3.42 [0.57]	-	
Missing	80	-	
Patient Health Questionnaire score			
Presence of depression symptoms*	257 (21.9%)	124 (11.1%)	**<0.001**
Minimal /no depression symptoms	917 (78.1%)	996 (88.9%)	
Missing			
Generalised Anxiety Disorder Questionnaire Score			
Presence of anxiety symptoms*	172 (14.7%)	72 (6.4%) <0.001	
Minimal/no anxiety symptoms	1002 (85.3%)	1048 (93.6%)	
Missing			
Socioeconomic adversity			
Low asset index	283 (24.1%)	-	
Food insecurity	125 (11.0%)	-	
Low caregiver education	382 (32.5%)	-	

1 n (%); Mean [SD]

* Scores above the test cut-off (10 and higher)

## Data Availability

De-identified study data is available on request.
